# Oncogenic LMO3 Collaborates with HEN2 to Enhance Neuroblastoma Cell Growth through Transactivation of *Mash1*

**DOI:** 10.1371/journal.pone.0019297

**Published:** 2011-05-05

**Authors:** Eriko Isogai, Miki Ohira, Toshinori Ozaki, Shigeyuki Oba, Yohko Nakamura, Akira Nakagawara

**Affiliations:** 1 Division of Biochemistry and Innovative Cancer Therapeutics, Chiba Cancer Center Research Institute, Chuoh-ku, Chiba, Japan; 2 Laboratory of Cancer Genomics, Chiba Cancer Center Research Institute, Chuoh-ku, Chiba, Japan; 3 Laboratory of Anti-Tumor Research, Chiba Cancer Center Research Institute, Chuoh-ku, Chiba, Japan; 4 Integrated Systems Biology Laboratory, Department of Systems Science, Graduate School of Informatics, Kyoto University, Gokasho, Uji, Kyoto, Japan; Institute of Genetics and Molecular and Cellular Biology, France

## Abstract

Expression of *Mash1* is dysregulated in human neuroblastoma. We have also reported that LMO3 (LIM-only protein 3) has an oncogenic potential in collaboration with neuronal transcription factor HEN2 in neuroblastoma. However, the precise molecular mechanisms of its transcriptional regulation remain elusive. Here we found that LMO3 forms a complex with HEN2 and acts as an upstream mediator for transcription of *Mash1* in neuroblastoma. The high levels of *LMO3* or *Mash1* mRNA expression were significantly associated with poor prognosis in 100 primary neuroblastomas. The up-regulation of *Mash1* remarkably accelerated the proliferation of SH-SY5Y neuroblastoma cells, while siRNA-mediated knockdown of *LMO3* induced inhibition of growth of SH-SY5Y cells in association with a significant down-regulation of *Mash1*. Additionally, overexpression of both LMO3 and HEN2 induced expression of *Mash1*, suggesting that they might function as a transcriptional activator for *Mash1*. Luciferase reporter assay demonstrated that the co-expression of LMO3 and HEN2 attenuates HES1 (a negative regulator for *Mash1*)-dependent reduction of luciferase activity driven by the *Mash1* promoter. Chromatin immunoprecipitation assay revealed that LMO3 and HEN2 reduce the amount of HES1 recruited onto putative HES1-binding sites and E-box within the *Mash1* promoter. Furthermore, both LMO3 and HEN2 are physically associated with HES1 by immunoprecipitation assay. Thus, our present results suggest that a transcriptional complex of LMO3 and HEN2 may contribute to the genesis and malignant phenotype of neuroblastoma by inhibiting HES1 which suppresses the transactivation of *Mash1*.

## Introduction

Neuroblastoma is one of the typical childhood cancers and is originated from sympathetic cell lineage of the neural crest [1], [2]. Since the tumor never occurs from the other lineages of the neural crest, the oncogenic events to cause neuroblastoma might be strictly regulated in a lineage-specific manner [1], [2].

LIM-only protein (LMO) family is composed of four members, LMO1, LMO2, LMO3 and LMO4. Although LMO proteins lack a DNA-binding activity, accumulating evidence suggest that LMO proteins are involved in transcriptional regulation of specific target genes in collaboration with other transcription factors [3]. Genetic analyses demonstrated that LMO1 and LMO2 contribute to the genesis of immature and aggressive T-cell leukemia [4], whereas LMO4 was implicated in development of breast cancer [5], [6]. Previously, we reported that *LMO3* is expressed at significantly high levels in human unfavorable neuroblastomas relative to favorable ones, and has an oncogenic potential in neuroblastoma [7]. LMO3 formed a complex with neuronal-specific basic helix-loop-helix (bHLH) transcription factor HEN2, which was also expressed at higher levels in unfavorable neuroblastoma than favorable one, raising a possibility that LMO3 may form a complex with HEN2 and play an important role in genesis and development of neuroblastoma through transcriptional regulation of as yet unidentified target gene(s).

A proneural bHLH transcription factor termed Mash1 plays a critical role in development of sympathetic neuron and is highly expressed in neuroblastoma [8], [9]. However, its possible contribution to development of neuroblastoma remains elusive. A bHLH protein termed HES1 acts as a negative regulator for *Mash1* [10]. Intriguingly, studies in *Drosophila* demonstrated that expression levels of *achaete-scute*, a *Drosophila* homolog of *Mash1*, are remarkably induced by a transcriptional complex composed of *Drosophila* homolog of LMO (dLMO) and bHLH proteins [11], [12].

In this study, we examined whether there could exist functional relationship between LMO3/HEN2 and Mash1 in neuroblastoma, and found that LMO3/HEN2 attenuates HES1 function and enhances transactivation of *Mash1*, leading to aggressive phenotype of neuroblastoma.

## Results

### High levels of *Mash1* expression is associated with poor outcome of neuroblastoma

**Figure 1:**
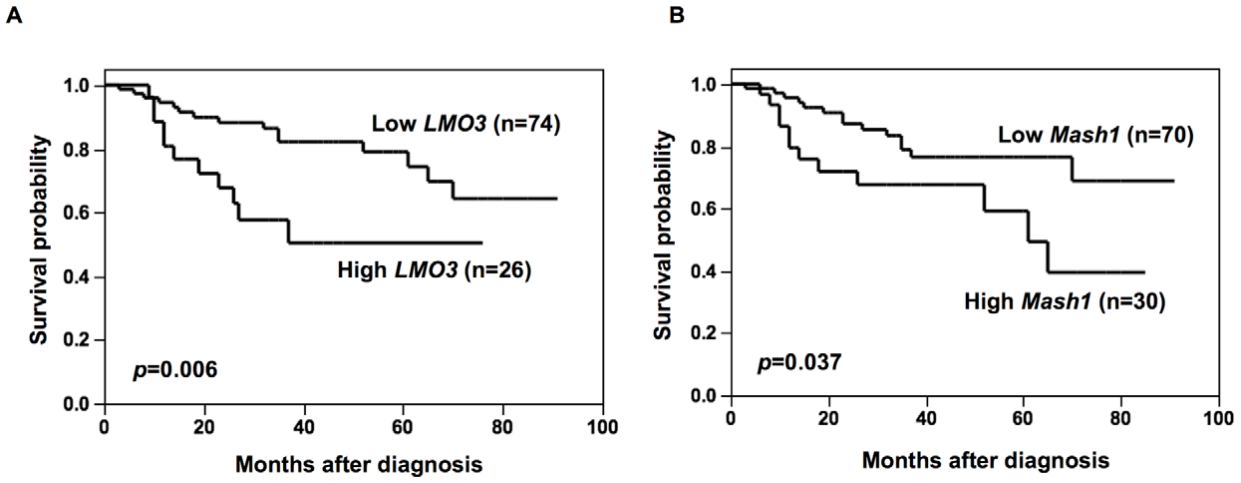
Kaplan-Meier survival curves of patients with neuroblastomas based on high or low expression of *LMO3* (A) or *Mash1* (B). Kaplan-Meier survival curves (n = 100) in relation to the expression levels of *LMO3* or *Mash1* (average cutoff). The patients with high expression of *LMO3* or *Mash1* represented significantly poor prognosis than those with its low expression.

*Mash1* is constitutively expressed at high levels in neuroblastoma cell lines and primary neuroblastoma tumors [9], [13], however, its prognostic significance remained elusive. On the other hand, expression of *LMO3* was significantly associated with poor outcome of the patients [7]. To verify whether a significant relationship could be observed between expression of *LMO3* and that of *Mash1* in primary neuroblastomas, we quantitatively measured the expression levels of *LMO3* and *Mash1* mRNA in 100 primary tumors by using a quantitative real-time RT-PCR. The student's t-test showed that high expression of *LMO3* was significantly associated with ≥1 year of age (*p* = 0.036), low expression of *TrkA* (*p* = 0.003) and *MYCN* amplification (*p* = 0.04), but not with the tumor stage (*p* = 0.17), tumor origin (*p* = 0.083) and Shimada classification (*p* = 0.082). High expression of *Mash1* was significantly associated with advanced tumor stage (*p* = 0.004) but not with age (*p* = 0.81), *TrkA* expression (*p* = 0.4), *MYCN* copy number (*p* = 0.11), tumor origin (*p* = 0.2) and Shimada classification (*p* = 0.45) (Table S1). No significant relationship was observed between *LMO3* and *Mash1* mRNA expression levels (the Pearson correlation coefficient was 0.27). Kaplan-Meier survival curves indicated that high expression of *LMO3* as well as that of *Mash1* were significantly associated with poor prognosis (log-rank test, *p* = 0.006 and *p* = 0.037, respectively; Figure 1). The univariate analysis according to the Cox proportional hazard model also indicated that the expression levels of *Mash1* and those of *LMO3* were significantly associated with poor outcome of the patients (*p* = 0.048 and *p* = 0.012, respectively; Table S2). The multivariate Cox proportional hazard model analysis showed that the expression of *Mash1* was significantly independent prognostic factor from *LMO3* expression and age, marginally from *MYCN* copy number and origin, but not from the disease stage, and that the expression of *LMO3* was significantly independent prognostic factor from *Mash1* expression, age, the disease stage and origin, but not from *MYCN* copy number (Table S2). Thus, the results obtained from the primary neuroblastomas suggested that both high mRNA expression of *LMO3* and *Mash1* were strongly associated with poor prognoses of the patients with neuroblastoma but the way of contribution of those seemed to be rather independent.

### *Mash1* mediates growth promotion of neuroblastoma cells

**Figure 2:**
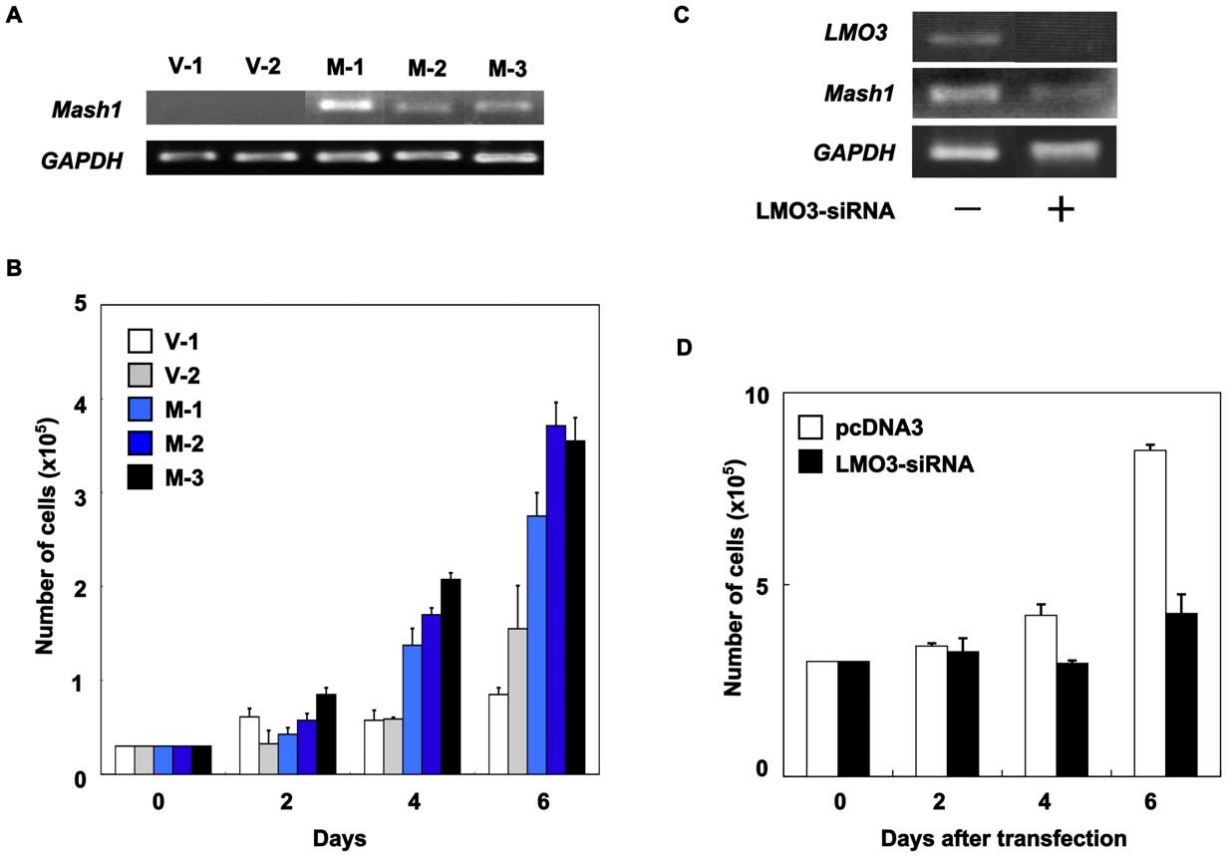
Mash1-mediated growth promotion of neuroblastoma cells. (A) Enforced expression of *Mash1*. Neuroblastoma SH-SY5Y cells were infected with empty retrovirus or with retrovirus encoding Mash1 and established two control infectants (V-1 and V-2) and three infectants expressing *Mash1* (M-1, M-2 and M-3). Total RNA was extracted from the indicated cell clones and subjected to RT-PCR to examine expression levels of *Mash1*. *GAPDH* was used as an internal control. (B) Mash1-mediated growth promotion. The indicated infectants were seeded at a density of 3×10^4^/cell culture dish and allowed to attach overnight. At the indicated time periods, number of viable cells was measured. (C) siRNA-mediated knockdown of LMO3. SH-SY5Y cells were transfected with empty plasmid (4 µg) or with expression plasmid for siRNA targeting LMO3 (4 µg). Forty-eight hours after transfection, total RNA was prepared and analyzed for expression levels of *LMO3* and *Mash1* by RT-PCR. (D) Decreased growth rate in LMO3-knocked down cells. SH-SY5Y cells (3×10^5^ cells/cell culture dish) were transfected as in (C). Forty-eight hours after transfection, cells were transferred into fresh medium. At the indicated time points, number of viable cells was measured.

Since *Mash1* is highly expressed in primary neuroblastoma [9] and its higher expression was significantly correlated with poor prognosis of the patient with neuroblastoma, we then investigated a possible contribution of Mash1 to neuroblastoma cell growth. For this purpose, we established three stable *Mash1* infectants derived from the parental SH-SY5Y neuroblastoma cells expressing exogenous Mash1 (M-1, M-2 and M-3) and two control vector alone infectants (V-1 and V-2) by retrovirus-mediated gene transfer (Figure 2A). As shown in Figure 2B, constitutive expression of *Mash1* in SH-SY5Y cells resulted in a remarkable increase in their growth rate as compared with the control infectants, suggesting that Mash1 is involved in regulation of neuroblastoma cell growth.

As described previously [7], LMO3 has an oncogenic potential in collaboration with HEN2 in neuroblastoma cells. We then asked whether or not LMO3 is involved in the Mash1-mediated enhancement of cell growth. As shown in Figure 2C, siRNA-mediated knockdown of *LMO3* in SH-SY5Y cells was significantly associated with a down-regulation of *Mash1*. Additionally, LMO3-knocked down SH-SY5Y cells showed a slower growth rate than the control SH-SY5Y cells (Figure 2D), which might be at least in part due to reduction of *Mash1*. We conducted the same experiments by using another cell line SK-N-BE and obtained the similar results (Figure S1A and B). We then hypothesized that *Mash1* could be one of transcriptional targets of LMO3/HEN2 complex.

### LMO3/HEN2 mediate transcriptional induction of *Mash1*

**Figure 3:**
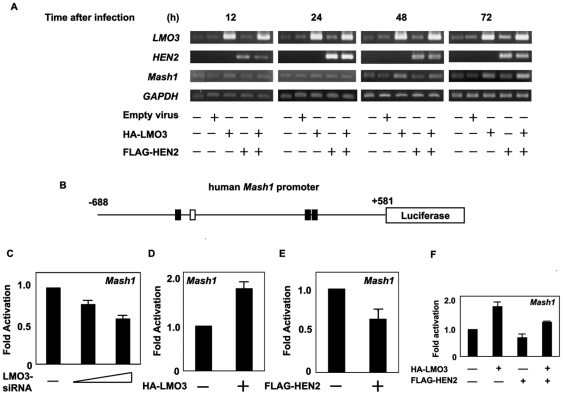
LMO3/HEN2-mediated transcriptional induction of *Mash1*. (A) RT-PCR. SH-SY5Y cells were infected with empty adenovirus or with the indicated combinations of recombinant adenovirus encoding HA-LMO3 or FLAG-HEN2. At the indicated time points after infection, total RNA was analyzed for expression levels of *LMO3*, *HEN2* and *Mash1* by RT-PCR. *GAPDH* was used as an internal control. (B) Schematic drawing of human *Mash1* promoter. Nucleotide positions were indicated relative to transcriptional initiation site (+1). The putative HES1-binding sites and E-box were depicted by filled and open boxes, respectively. This genomic fragment was subcloned into appropriate restriction sites of pGL3-Basic Vector to give pluc-hMash1. (C) siRNA-mediated knockdown of LMO3 reduces the promoter activity of *Mash1*. SH-SY5Y cells were co-transfected with constant amount of pluc-Mash1 (100 ng) and pRL-CMV (0.2 ng) in the presence or absence of increasing amounts of expression plasmid for siRNA against human LMO3 (100 or 400 ng). Forty-eight hours after transfection, cells were lysed and their luciferase activities were measured. (D) LMO3 transactivates *Mash1* promoter. Mouse neuroblastoma Neuro2a cells (1×10^5^ cells/24-well plate) were co-transfected with constant amount of pluc-hMash1 (100 ng) and pRL-CMV (0.2 ng) together with or without expression plasmid for HA-LMO3 (150 ng). Forty-eight hours after transfection, cells were lysed and their luciferase activities were measured. (E) HEN2 inhibits *Mash1* promoter activity. Luciferase activities were measured in Neuro2a cells with or without FLAG-HEN2 (100 ng). (F) LMO3 interferes with negative effect of HEN2 on Mash1 transcription in Neuro2a cells. Luciferase activities were measured in Neuro2a cells transfected with HA-LMO3 (150 ng), FLAG-HEN2 (100 ng) or both of them.

To address whether *Mash1* transcription could be induced by LMO3/HEN2, SH-SY5Y cells were infected with the indicated combinations of recombinant adenoviruses encoding HA-LMO3 or FLAG-HEN2, and the expression levels of *Mash1* were examined by semi-quantitative RT-PCR. Time course experiments demonstrated that *Mash1* is readily detectable in cells expressing HA-LMO3 alone or in cells co-expressing with HA-LMO3 and FLAG-HEN2 at 48 h after infection (Figure 3A). Seventy-two hours after infection, co-expression of HA-LMO3 and FLAG-HEN2 led to a significant induction of *Mash1*. The induction of *Mash1* was also observed in SK-N-BE cells transfected with expression vector HA-LMO3 alone or HA-LMO3 and FLAG-HEN2 at 72 h after transfection (Figure S1C). To further confirm these observations, we generated a luciferase reporter construct carrying human *Mash1* promoter (pluc-Mash1). As shown in Figure 3B, the 5′-upstream region of *Mash1* gene contains three putative HES1-binding sites and one E-box. In both SH-SY5Y cells and SK-N-BE cells, siRNA-mediated knockdown of human *LMO3* reduced promoter activity of *Mash1* in a dose-dependent manner (Figure 3C and Figure S1D). For luciferase reporter assay without siRNA for human LMO3, we used mouse neuroblastoma Neuro2a cells which displayed higher transfection efficiency than human neuroblastoma cells as examined by GFP staining (data not shown). Consistent with the above expression studies, LMO3 enhanced luciferase activity driven by *Mash1* promoter (Figure 3D). Furthermore, we examined the effect of HEN2 on *Mash1* promoter activity in Neuro2a cells, showing that HEN2 itself inhibited *Mash1* promoter activity (Figure 3E). Intriguingly, however, LMO3 interfered with HEN2 function, resulting in up-regulation of *Mash1* transcription (Figure 3F). Thus, it is likely that the LMO3 complex including HEN2 and HES1 regulates transcription of *Mash1*. The mRNA expression pattern of *LMO3*, *HEN2*, *Mash1* and *HES1*, a negative regulator of *Mash1* transcription, in neuroblastoma cell lines is shown in Figure S2.

### LMO3/HEN2 attenuates HES1-dependent down-regulation of *Mash1*

**Figure 4:**
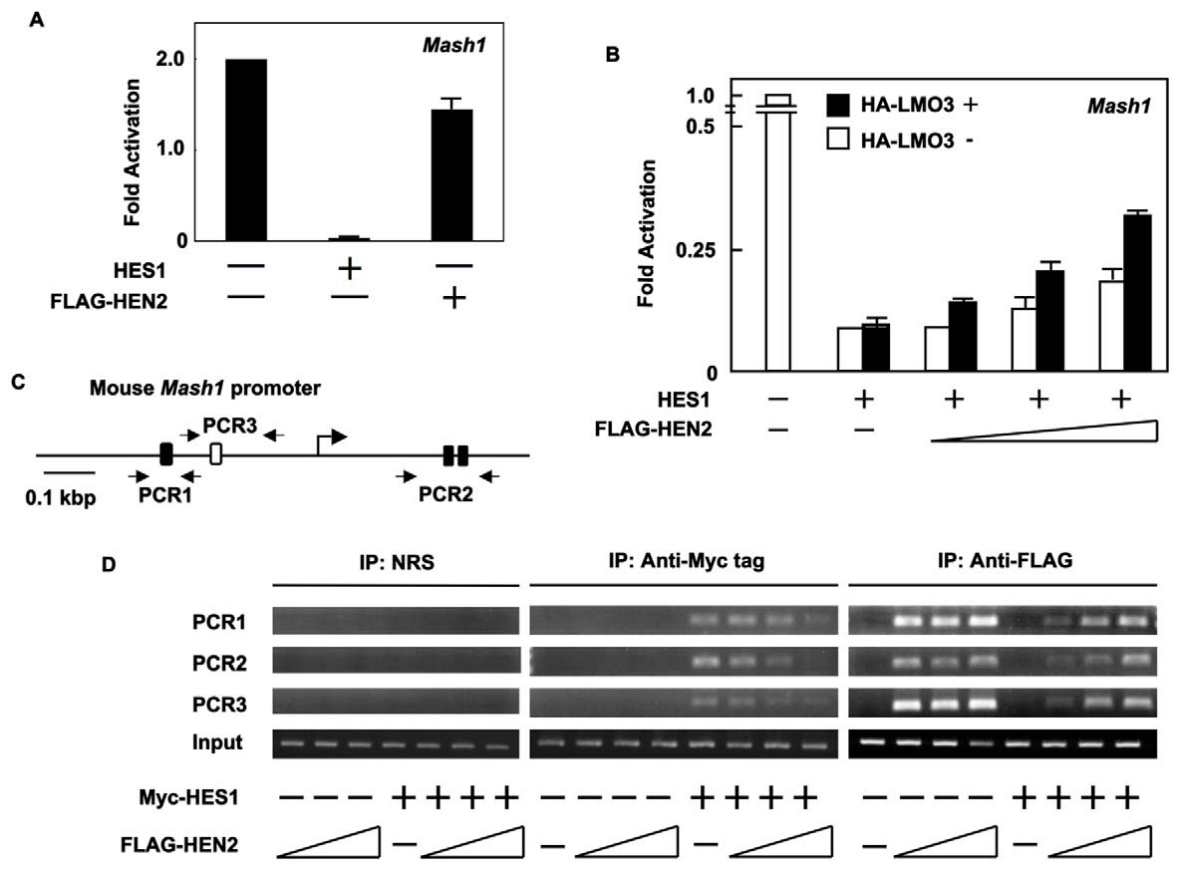
LMO3/HEN2 attenuates HES1-dependent down-regulation of *Mash1*. (A) Luciferase reporter assay. Neuro2a cells were co-transfected with constant amount of pluc-hMash1 (100 ng), pRL-CMV (0.2 ng) and expression plasmid for HES1 (50 ng) or HEN2 (50 ng). Forty-eight hours after transfection, cells were lysed and their luciferase activities were examined. (B) Luciferase reporter assay. Neuro2a cells were co-transfected with constant amount of pluc-hMash1 (100 ng), pRL-CMV (0.2 ng) and expression plasmid for HES1 (5 ng) in the presence or absence of expression plasmid for HA-LMO3 (150 ng) together with or without increasing amounts of FLAG-HEN2 expression plasmid (100, 200 or 300 ng). Forty-eight hours after transfection, cells were lysed and their luciferase activities were examined. (C) Schematic representation of mouse *Mash1* promoter. The canonical HES1-binding sites and E-box were indicated by filled and open boxes, respectively. The positions of primer sets used for chromatin immunoprecipitation (ChIP) assays were also indicated. (D) ChIP assay. Cross-linked chromatin prepared from Neuro2a cells transfected with the indicated combinations of expression plasmids was sonicated and immunoprecipitated with normal rabbit serum (NRS), polyclonal anti-Myc tag or with polyclonal anti-FLAG antibody. The genomic DNA was purified from the immunoprecipitates and amplified by PCR.

As reported previously [10], HES1 is one of the negative regulators for *Mash1*. In accordance with the previous observations, enforced expression of HES1 dramatically reduced luciferase activity driven by *Mash1* promoter (Figure 4A). The inhibitory effect of HES1 on *Mash1* promoter was stronger than that of HEN2. To investigate the relationships between HES1 and LMO3/HEN2 in transcriptional regulation of *Mash1*, we examined effects of HEN2 and LMO3 on HES1-dependent down-regulation of *Mash1* (Figure 4B). The HES1-dependent inhibition of *Mash1* promoter activity was attenuated by co-expression with FLAG-HEN2 alone or with co-expression with FLAG-HEN2 plus HA-LMO3. Inhibitory effects of FLAG-HEN2 plus HA-LMO3 on HES1 were larger than that of FLAG-HEN2 alone, suggesting that LMO3/HEN2 complex plays a critical role in regulation of *Mash1* transcription by neutralizing the inhibitory effect of HES1.

To ask about mechanistic insights into understanding how LMO3 and/or HEN2 could attenuate the inhibitory effects of HES1 on *Mash1* expression, we performed chromatin immunoprecipitation (ChIP) assay. Similar to human *Mash1* promoter, mouse *Mash1* promoter also contains three putative HES1-binding sites and one E-box (Figure 4C). Neuro2a cells were transfected with constant amount of empty plasmid or with expression plasmid for Myc-HES1 together with or without increasing amounts of FLAG-HEN2 expression plasmid. Forty-eight hours after transfection, cross-linked chromatin was prepared and subjected to ChIP assay. As shown in Figure 4D, the anti-Myc tag immunoprecipitates contained genomic fragments including putative HES1-binding sites as well as E-box. The amounts of Myc-HES1 recruited onto HES1-binding sites and E-box significantly decreased in the presence of FLAG-HEN2 in a dose-dependent manner. Additionally, the anti-FLAG immunoprecipitates contained genomic fragments including putative HES1-binding sites and E-box in the absence of exogenous HES1. Co-expression of FLAG-HEN2 and Myc-HES1 inhibited recruitment of FLAG-HEN2 onto putative HES1-binding sites and E-box, however, its inhibition was efficiently abrogated by increasing amounts of FLAG-HEN2. These results suggest that HEN2 might compete with HES1 in binding to putative HES1-binding sites and E-box, and thereby inducing the expression of *Mash1*.

### HEN2 Interacts with HES1 in cells

**Figure 5:**
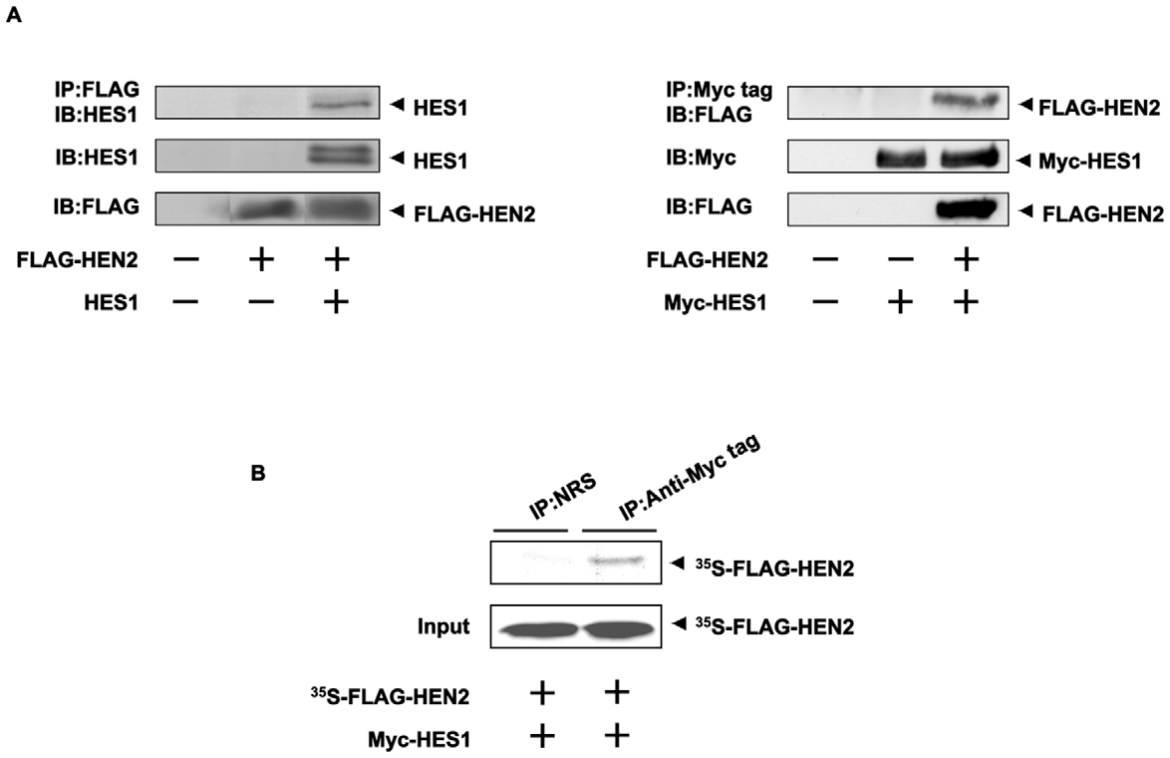
Interaction between HEN2 and HES1 in cells. (A) Neuro2a cells were co-transfected with the indicated combinations of expression plasmids. Forty-eight hours after transfection, cells were lysed and immunoprecipitated with anti-FLAG (left panel) or with anti-Myc tag antibody (right panel) and the immunoprecipitates were analyzed by immunoblotting with anti-HES1 or with anti-FLAG antibody, respectively. Aliquots of cell lysates were subjected to immunoblotting with anti-HES1, anti-FLAG or with anti-Myc tag antibody. (B) *In vitro* pull-down assay. Radio-labeled FLAG-HEN2 was incubated with cell lysates prepared from Neuro2a cells transfected with Myc-HES1 expression plasmid. The reaction mixture was immunoprecipitated with normal rabbit serum (NRS) or with polyclonal anti-Myc tag antibody and separated by SDS-PAGE followed by autoradiography. 1/5 inputs were also shown.

**Figure 6:**
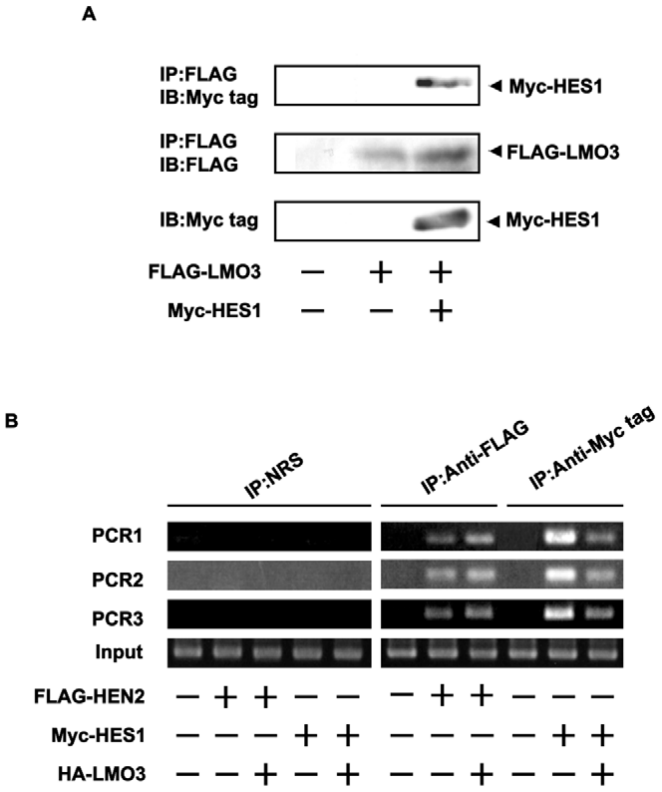
LMO3 attenuates binding of HES1 to Mash1 promoter and promotes that of HEN2. (A) Complex formation between LMO3 and HES1 in cells. Neuro2a cells were transiently transfected with the indicated combinations of the expression plasmids. Forty-eight hours after transfection, cell lysates were immunoprecipitated with anti-FLAG antibody followed by immunoblotting with anti-Myc tag antibody (top panel). Expressions of FLAG-LMO3 and Myc-HES1 are also shown (lower panels). (B) ChIP assay. Cross-linked chromatin prepared from Neuro2a cells transfected with the indicated combinations of expression plasmids was sonicated and immunoprecipitated with normal rabbit serum (NRS), polyclonal anti-Myc tag or with polyclonal anti-FLAG antibody. The genomic DNA was purified from the immunoprecipitates and amplified by PCR.

**Figure 7:**
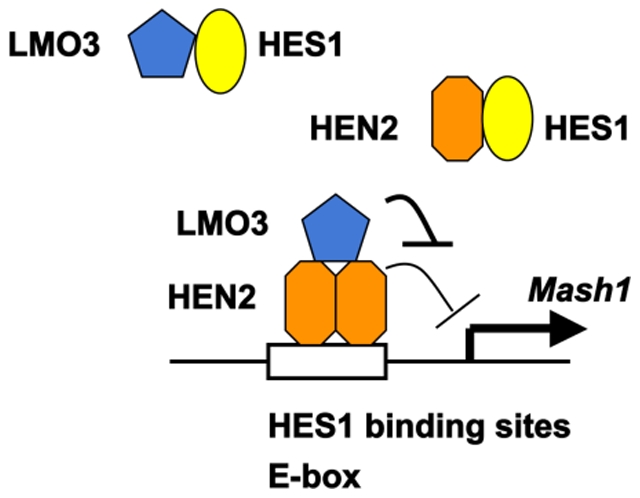
Model for LMO3 and HEN2 cooperation in transcriptional regulation of Mash1 in Neuroblastoma. HES1 binds to HES1 binding sites and E-box on *Mash1* promoter and represses *Mash1* transcription. LMO3 inhibits recruitment of HES1 onto HES1-binding sites and E-box on *Mash1* promoter by forming complex with HES1. HEN2 interferes with recruitment of HES1 onto HES1-binding sites and E-box on *Mash1* promoter by forming complex with HES1 and competing with HES1 in binding to these sites. LMO3 promotes recruitment of HEN2 onto HES1-binding sites and E-box on *Mash1* promoter by forming complex with HEN2 but inhibits negative effects of HEN2 on *Mash1* promoter. Thereby expression of *Mash1* is up-regulated.

To examine whether HEN2 could interact with HES1 in cells, we performed immunoprecipitation experiments. Cell lysates prepared from Neuro2a cells co-transfected with the indicated combinations of expression plasmids were subjected to immunoprecipitation. As clearly shown in Figure 5A, HES1 was co-immunoprecipitated with FLAG-HEN2. Consistent with these results, reciprocal experiments showed that the anti-Myc tag immunoprecipitates contain FLAG-HEN2. *In vitro* pull-down assay demonstrated that radio-labeled FLAG-HEN2 is co-immunoprecipitated with Myc-HES1 (Figure 5B). Additional immunoprecipitation experiments demonstrated that LMO3 also forms a stable complex with HES1 (Figure 6A). We have previously showed that LMO3 forms a stable complex with HEN2 [7]. To investigate the effect of LMO3 on binding of HEN2 and HES1 to putative HES1-binding sites and E-box, Neuro2a cells were transfected with Myc-HES1 or FLAG-HEN2 together with or without HA-LMO3 expression plasmid and subjected to ChIP assay. As shown in Figure 6B, the immunoprecipitates using anti-Myc tag or anti-FLAG tag antibody contained genomic fragments including putative HES1-binding sites as well as E-box. The amount of Myc-HES1 recruited onto HES1-binding sites and E-box decreased in the presence of HA-LMO3. On the other hand, the amount of FLAG-HEN2 recruited onto HES1-binding sites and E-box increased in the presence of HA-LMO3. As shown in Figure 3F, LMO3 interferes with inhibitory effect of HEN2 on Mash1 expression. These suggest that LMO3 may additively interfere with the inhibitory effect of HES1 on *Mash1* expression by promoting binding of HEN2 to HES1-binding sites and E-box. Collectively, it is conceivable that LMO3/HEN2 reduces the inhibitory effect of HES1 on *Mash1* expression through binding to HES1 and thereby blocking its recruitment onto putative HES1-binding sites and E-box (Figure 7 and Figure S3).

## Discussion

In this study, we found that *Mash1* is one of transcriptional targets of LMO3/HEN2 transcriptional complex, and its protein product may play an important role in regulation of neuroblastoma cell growth. As described previously [14], *HEN1* as well as its closely related gene *HEN2* encodes bHLH-type transcription factor, which might recognize E-box (5′-CACGTG-3′). On the other hand, HES1 has an intrinsic transcriptional repressor activity [10]. Based on our present results, adenovirus-mediated expression of LMO3/HEN2 significantly induced *Mash1*, and HES1-mediated down-regulation of *Mash1* promoter activity was recovered by co-expression of LMO3 and HEN2. Our ChIP analyses indicated that HES1 binds to HES1-recognition sites and E-box within *Mash1* promoter in the absence of HEN2, whereas HEN2 efficiently inhibits the recruitment of HES1 onto HES1-binding sites and E-box within *Mash1* promoter, suggesting that HES1 occupies HES1-binding sites and E-box to inhibit the promoter activity of *Mash1*. On the other hand, HEN2 formed a complex with HES1 and reduced the amounts of HES1 recruited onto HES1-binding sites as well as E-box to increase the promoter activity of *Mash1* in collaboration with LMO3. Thus, it is likely that the balance between intracellular amounts of HES1 and LMO3/HEN2 might determine expression levels of *Mash1*, and thereby regulating neuroblastoma cell growth.

It was reported that de-repression of *Mash1* might interfere with differentiation of sympatho-adrenal precursors of *Insm1* mutant mice although *Mash1* is expressed transiently in those cells during normal neural differentiation [15]. Furthermore, Watt et al. reported that N-myc positively regulates *Mash1* transcription [16]. Therefore, it is possible that in the transcriptional regulation of *Mash1*, LMO3 and HEN2 may associate with other nuclear factors like Insm1 and N-myc besides HES1.

From the developmental point of view, it is known that the LMO/HEN complex plays an important role in regulating neuronal differentiation [11], [17]. As described [7], [18], expression of *LMO3* was highly restricted in adult and fetal brains, and *HEN2* was expressed in developing nervous system. Genetic studies demonstrated that HEN2 participates in proper neural crest-derived neuroendocrine development and that Mash1 has a critical role in maintaining neuroendocrine cell phenotype [19], [20]. Although *LMO3*-knockout mice did not exhibit any significant developmental defects, mice lacking both LMO1 and LMO3 died after birth, which might be due to neural defects [21]. Since neuroblastoma is one of the most common childhood solid tumors of peripheral nervous system arising from as yet unidentified population of neural crest cells [22] and Mash1 regulates proliferation of the sympathetic nervous system [23], it is likely that deregulated expression of Mash1 could contribute to genesis and development of neuroblastoma, which might be regulated by LMO3/HEN2 transcriptional complex both *in vitro* and *in vivo*. This LMO3/HEN2-HES1-Mash1 pathway could be the new future target for developing the anti-neuroblastoma treatment.

## Materials and Methods

### Ethics Statement

A hundred human neuroblastoma specimens used in the present study were kindly provided from various institutions and hospitals in Japan to the Chiba Cancer Center Neuroblastoma Tissue Bank. Written informed consent was obtained at each institution or hospital. This study was approved by the Chiba Cancer Center Institutional Review Board and were conducted according to the principles expressed in the Declaration of Helsinki.

### Tumor Specimens

Tumors were classified according to the International Neuroblastoma Staging System (INSS); 25 Stage 1, 13 Stage 2, 33 Stage 3, 23 Stage 4, and 6 Stage4s. Clinical information including age at diagnosis, tumor origin, Shimada's histology, prognosis, and survival months of each patient were obtained. The median follow-up time for survivors was 35 months (range 3 to 91 months). Each tumor specimen was assayed for *TRKA* expression by Northern blot analysis and for *MYCN* amplification status by both fluorescence in situ hybridization (FISH) and real-time quantitative polymerase chain reaction (PCR).

### Quantitative Real-time PCR

Total RNA prepared from primary neuroblastomas was reverse transcribed into cDNA (SuperScript II kit) and subjected to the real-time PCR. The expression level of *GAPDH* was measured in all samples to normalize *LMO3* and *Mash1* expression according to the manufacturer's instructions (Applied Biosystems, Foster City, CA, USA). Oligonucleotide primers and TaqMan probes, which were labeled at the 5′ end with the reporter dye 6-carboxyfluorescein (FAM) and at the 3′ end with the quencher dye 6-carboxytetramethylrhodamine (TAMRA), were as follows: *LMO3*: forward 5′-TCTGAGGCTCTTTGGTGTAACG-3′, reverse 5′-CCAGGTGGTAAACATTGTCCTTG-3′ and probe 5′-FAM-AAACTGCGCTGCCTGTAGTAAGCTCATCC-TAMRA-3′. Taqman(R) Gene Expression Assay (Applied Biosystems) was purchased for *Mash1* with Assay ID Hs00269932-m1. Amplification and detection were done using the ABI Prism 7700 Sequence Detection System (Applied Biosystems).

### Statistical Analysis

Student's *t* tests were used to explore possible associations between *LMO3* expression and other factors. The distinction between high and low levels of *LMO3* and *Mash1* expression was based on the mean value. Kaplan-Meier survival curves were calculated, and survival distributions were compared using the log-rank test. Cox regression models were used to explore associations among *LMO3* expression, *Mash1* expression, age, *MYCN* amplification, tumor origin, Shimada classification and survival. Statistical significance was declared if *P*<0.05.

### Cell Culture and Transfection

SH-SY5Y (human neuroblastoma, ATCC number CRL-2266), SK-N-BE (human neuroblastoma, ATCC number CRL-2271) and Neuro2a (mouse neuroblastoma, ATCC number CCL-131) cells were maintained in RPMI 1640 supplemented with 10% heat-inactivated fetal bovine serum at 37°C in an atmosphere of 5% CO_2_ in the air. Cells were transfected with the indicated expression plasmids using Lipofectamine 2000 transfection reagent (Invitrogen, Carlsbad, CA, USA) as recommended by the manufacturer.

### Generation of Recombinant Retroviral Vector and Retrovirus-mediated Gene Transfer

Human *Mash1* cDNA was subcloned into the *Hpa*I restriction site of the pLXSN vector. pLXSN or pLXSN- *Mash1* was transfected into the ϕ2 packaging cells, and SH-SY5Y cells (1×10^6^ cells) infected with virus-containing culture medium were cultured in the medium containing 500 µg/ml G418 (Sigma Chemical Co., St. Louis, MO, USA). Two weeks after the selection in G418, drug-resistant clones were isolated and allowed to proliferate in medium containing G418.

### Reverse Transcription-PCR Analysis

Total RNA was prepared from cultured cells by using the RNeasy Mini Kit (Qiagen, Valencia, CA, USA). Reverse transcription was carried out using random primers and SuperScript II (Invitrogen). Following the reverse transcription, the resultant cDNA was subjected to PCR-based amplification. PCR primers used were as follows: human *LMO3*, forward 5′-ATGCTCTCAGTCCAGCCAGA-3′ and reverse 5′-TCAGCGAACCTGGGGTGCAT-3′; human *HEN2*, forward 5′-AAGCAGCAGATTCGGACCAT-3′ and reverse 5′-CTTCTCCTCGCGGCTCAG-3′; human *Mash1*, forward 5′-GCGTTCAGCACTGACTTTTG-3′ and reverse 5′-CCCCGGGAGACTTCTTAGAG-3′; human *HES1*, forward 5′-TGAGCCAGCTGAAAACACTG-3′ and reverse 5′-GTCACCTCGTTCATGCACTC-3; human *glyceraldehydes-3-phosphate dehydrogenase* (*GAPDH*), forward 5′-ACCTGACCTGCCGTCTAGAA-3′ and reverse 5′-TCCACCACCCTGTTGCTGTA-3′.

### RNA Interference Experiments

Human LMO3 RNAi vector was made using the original plasmid that is gift from A.K. Munirajan (Chiba Cancer Center Research Institute). The targeted sequence is 5′- GTAGTAAGCTCATCCCTGC -3′. RNAi construct was transiently transfected into SH-SY5Y cells using Lipofectamine 2000 transfection reagent (Invitrogen, Carlsbad, CA, USA) according to the manufacturer's instruction.

### Generation of Recombinant Adenoviral Vector

For construction of the adenovirus expression vector, an HA-tagged human LMO3 cDNA or a FLAG-tagged human HEN2 cDNA were inserted into the shuttle vector pHMCMV6 [24]. Efficient construction of a recombinant adenovirus vector by an improved *in vitro* ligation method [25]. The resultant shuttle vector was digested with I-CeuI and PI-SceI and subcloned into the identical restriction sites of the adenovirus expression vector pAdHM4. The recombinant adenovirus construct was digested with *Pac*I and transfected into 293 cells to generate recombinant adenovirus.

### Luciferase Reporter Assay

The reporter plasmid contains a 1.2-kb fragment of the human *Mash1* promoter that was subcloned into the pGL3-Basic Vector (Promega Corp., Madison, WI, USA) upstream of the luciferase reporter gene. Cells were seeded in triplicates into 24-well plates (1×10^5^ cells/well) 24 h prior to transfection. Cells were cotransfected with 100 ng of the reporter plasmid, 0.2 ng of pRL-CMV encoding *Renilla* luciferase cDNA, 5 ng of rat HES1expression vector, 150 ng of HA-LMO3, 100 to 300 ng of FLAG-HEN2 expression vectors. Total amount of plasmid DNA per transfection was kept constant with pcDNA3 (Invitrogen). At 48 h after transfection, luciferase activity was measured by a Dual-Luciferase Reporter Assay System (Promega), and the transfection efficiency was standardized against *Renilla* luciferase activity.

### Chromatin Immunoprecipitation (ChIP) Assays

ChIP assay was performed according to the protocol recommended by Upstate (Lake Placid, NY, USA). Cross-linked chromatin prepared from Neuro2a cells transfected with expression plasmids was sonicated and immunoprecipitated with normal rabbit serum (NRS), polyclonal anti-Myc tag (Medical & Biological Laboratories, Nagoya, Japan) or with polyclonal anti-FLAG (Sigma, St. Louis, MO, USA) antibody. The genomic DNA was purified from the immunoprecipitates and amplified by PCR.

The primers used to amplify the mouse *Mash1* promoters were as follows: HES1 binding site (PCR-1), forward 5′-ATTTCTAGAGCCACCCCCTG-3′ and reverse 5′-TTGTTGCAGTGCGTGCGCC-3′; HES1 binding site (PCR-2), forward 5′-AGTGCGCTCGGCACTGACTT-3′ and reverse 5′-CGCGGTTGGCTTCGGGAGCC-3′; E-box (PCR-3), forward 5′-ATGGAGAGTTTGCAAGGAGC-3′ and reverse 5′-CAGCCCCACGCGCAGCCCTG-3′.

### Western Blot Analysis and Immunoprecipitation

After transfection, Neuro2a cells were placed on ice, washed twice with phosphate-buffered saline, and lysed in lysis buffer containing 25 mM Tris-HCl (pH 8.0), 137 mM NaCl, 2.7 mM KCl, 1% TritonX-100, 1 mM phenylmethylsulfonyl fluoride and protease inhibitor mixture (Sigma). Lysates were placed on ice for 30 min, sonicated briefly, and clarified by centrifugation at 15,000× *g* for 5 min at 4°C. Protein concentrations of the supernatants were determined by using a Bio-Rad protein assay. For immunoblot analysis, proteins were resolved by sodium dodecyl sulfatepolyacrylamide gel electrophoresis (SDS-PAGE) and electrotransferred onto a nitrocellulose membrane. The membrane filter was blocked with 2% gelatin in Tris-buffered saline (TBS) for 3 h at room temperature and then incubated with a primary antibody including monoclonal anti-rat HES1 (Medical & Biological Laboratories, Nagoya, Japan), monoclonal anti-FLAG (M2; Sigma) or monoclonal anti-Myc (9B11; Cell Signaling Technology, Danvers, MA, USA) antibody over night at 4°C. The membrane filter was then incubated with a goat anti-mouse secondary antibody conjugated to horseradish peroxidase (Cell Signaling Technology, Danvers, MA, USA) or goat anti-rat secondary antibody conjugated to horseradish peroxidase (Beckman Coulter, Marseille, France) for 1 h at room temperature and bound secondary antibody was detected by enhanced chemiluminescence (Amersham Pharmacia Biotech) according to the manufacturer's protocol. For Immunoprecipitation, equal amounts of cell lysates (2 mg) were precleared with 25 µl of protein G-Sepharose (Amersham Bioscience, Uppsala, Sweden). After brief centrifugation, immunoprecipitation was carried out by incubating the supernatant with anti-FLAG polyclonal (Sigma, St. Louis, MO, USA) or anti-Myc tag polyclonal antibody (Medical & Biological Laboratories, Nagoya, Japan) over night at 4°C. Immunocomplexes were precipitated with protein G-Sepharose beads (Amersham Biosciences) for 3 hours at 4°C. The immunoprecipitated proteins were resolved by SDS-PAGE and analyzed by Western blotting.

### *In vitro* Pull-down Assay

Radio-labeled FLAG-HEN2 was generated by using *in vitro* transcription/translation system (Promega) and incubated with cell lysates prepared from Neuro2a cells transfected with Myc-HES1 expression plasmid. The reaction mixture was immunoprecipitated with normal rabbit serum (NRS) or with polyclonal anti-Myc tag antibody (Medical & Biological Laboratories, Nagoya, Japan) and separated by SDS-PAGE followed by autoradiography.

### Data Analysis and Statistics

All values for statistical significance represent mean ± SD. We carried out comparisons between means using the Student's *t*-test. Statistical significance implies *P*<0.05.

## Supporting Information

Figure S1 **Mash1-mediated growth promotion and LMO3/HEN2-mediated transcriptional induction of** ***Mash1*** **in SK-N-BE cells.** (A) siRNA-mediated knockdown of LMO3. SK-N-BE cells were transfected with empty plasmid (4 µg) or with expression plasmid for siRNA targeting LMO3 (4 µg). Forty-eight hours after transfection, total RNA was prepared and analyzed for expression levels of *LMO3* and *Mash1* by RT-PCR. (B) Decreased growth rate in LMO3-knocked down cells. SK-N-BE cells (4.5×10^3^ cells/well, 96 well culture plate) were transfected with empty plasmid (0.2 µg) or with expression plasmid for siRNA targeting LMO3 (0.2 µg). Forty-eight hours after transfection, cells were transferred into fresh medium. At the indicated time points, cell growth was measured by MTT assay (Cell Counting Kit-8, DOJINDO). (C) RT-PCR. SK-N-BE cells were transfected with pcDNA3 empty plasmid or with the indicated combinations of expression plasmid HA-LMO3 or FLAG-HEN2. At 72 hours after transfection, total RNA was analyzed for expression levels of *LMO3*, *HEN2* and *Mash1* by RT-PCR. *GAPDH* was used as an internal control. (D) siRNA-mediated knockdown of LMO3 reduces the promoter activity of *Mash1*. SK-N-BE cells were co-transfected with constant amount of pluc-Mash1 (100 ng) and pRL-CMV (0.2 ng) in the presence or absence of increasing amounts of expression plasmid for siRNA against human LMO3 (100 or 400 ng). Forty-eight hours after transfection, cells were lysed and their luciferase activities were measured.
(TIF)

Figure S2 **Expression of** ***LMO3*** **,** ***HEN2*** **,** ***Mash1*** **or** ***HES1*** **in neuroblastoma cell lines.** Semiquantitative RT-PCR analysis for expression of *LMO3*, *HEN3*, *Mash1* or *HES1* in neuroblastoma cell lines is performed under linear amplification conditions. Expression of *GAPDH* is shown as a control.
(TIF)

Figure S3 **Model for LMO3 and HEN2 cooperation in transcriptional regulation of** ***Mash1*** **in Neuroblastoma.** (A) HES1 binds to HES1 binding sites and E- box on *Mash1* promoter and represses *Mash1* transcription. (B) LMO3 inhibits recruitment of HES1 onto HES1-binding sites and E-box on *Mash1* promoter by forming complex with HES1, and thereby inducing the expression of Mash1. (C) HEN2 interferes with recruitment of HES1 onto HES1-binding sites and E-box on *Mash1* promoter by forming complex with HES1 and competing with HES1 in binding to these sites. HEN2 also represses *Mash1* transcription but the inhibitory effects are weaker than that of HES1, and so up-regulating transcription of *Mash1*. (D) LMO3 promotes recruitment of HEN2 onto HES1-binding sites and E-box on *Mash1* promoter by forming complex with HEN2 but inhibits negative effects of HEN2 on *Mash1* promoter. Furthermore, LMO3 inhibits recruitment of HES1 onto HES1-binding sites and E-box on *Mash1* promoter, and so *Mash1* may be more highly expressed.
(TIF)

Table S1 Correlation between expression of LMO3 or Mash1 and other prognostic factors (Student's *t*-test).
(PDF)

Table S2 Univariate and multivariate analyses of Mash1 and LMO3 mRNA expression as well as other prognostic factors in primary neuroblastomas.
(PDF)
