# Supplementary figures and images for: Oncogenic LMO3 Collaborates with HEN2 to Enhance Neuroblastoma Cell Growth through Transactivation of Mash1
Source: PLoS One. 2011 May 5;6(5):e19297. doi: 10.1371/journal.pone.0019297 (PMC3088666; doi:10.1371/journal.pone.0019297)

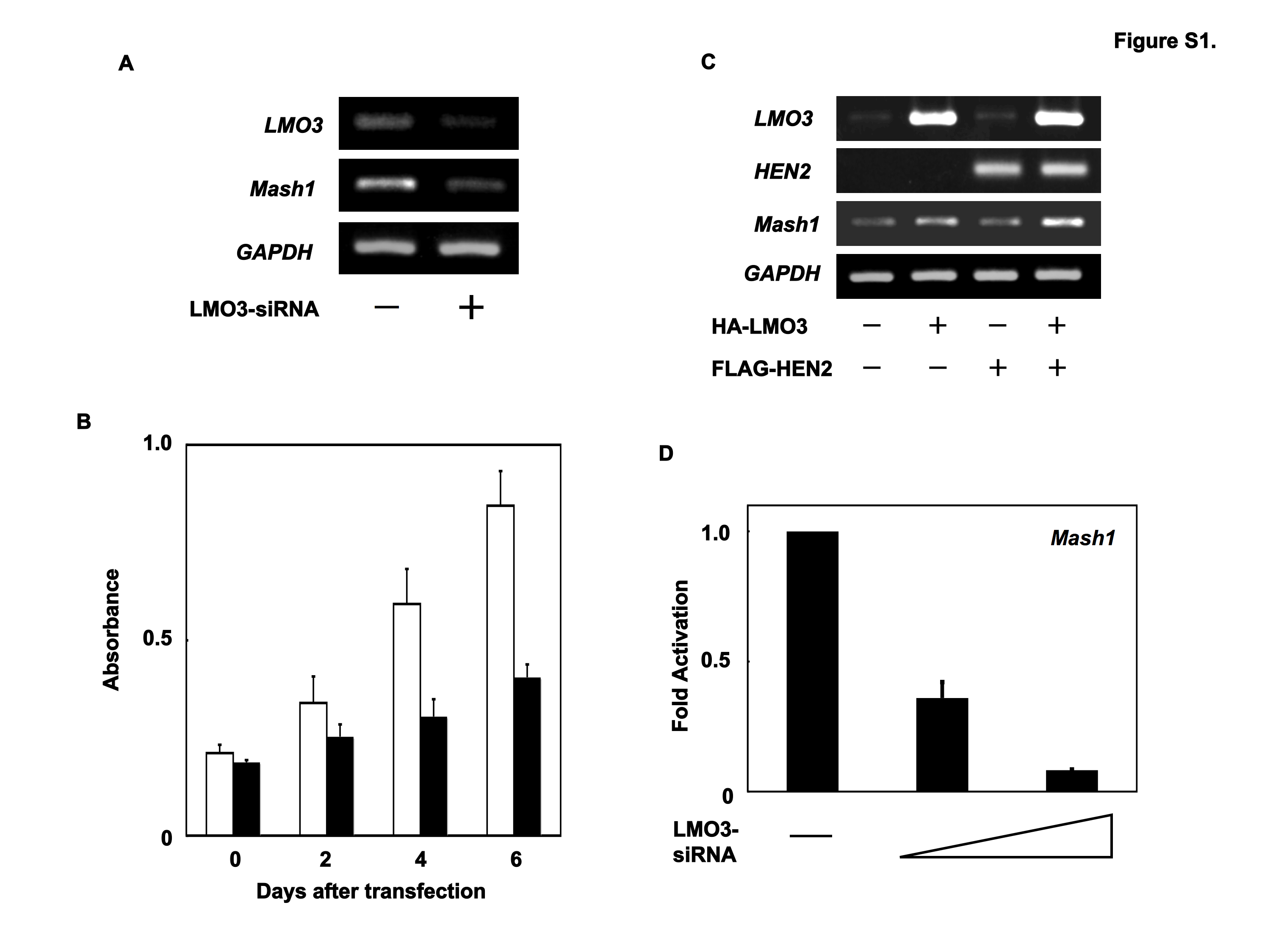

Supplement: Figure S1 — Mash1-mediated growth promotion and LMO3/HEN2-mediated transcriptional induction of Mash1 in SK-N-BE cells. (A) siRNA-mediated knockdown of LMO3. SK-N-BE cells were transfected with empty plasmid (4 µg) or with expression plasmid for siRNA targeting LMO3 (4 µg). Forty-eight hours after transfection, total RNA was prepared and analyzed for expression levels of LMO3 and Mash1 by RT-PCR. (B) Decreased growth rate in LMO3-knocked down cells. SK-N-BE cells (4.5×10^3^ cells/well, 96 well culture plate) were transfected with empty plasmid (0.2 µg) or with expression plasmid for siRNA targeting LMO3 (0.2 µg). Forty-eight hours after transfection, cells were transferred into fresh medium. At the indicated time points, cell growth was measured by MTT assay (Cell Counting Kit-8, DOJINDO). (C) RT-PCR. SK-N-BE cells were transfected with pcDNA3 empty plasmid or with the indicated combinations of expression plasmid HA-LMO3 or FLAG-HEN2. At 72 hours after transfection, total RNA was analyzed for expression levels of LMO3, HEN2 and Mash1 by RT-PCR. GAPDH was used as an internal control. (D) siRNA-mediated knockdown of LMO3 reduces the promoter activity of Mash1. SK-N-BE cells were co-transfected with constant amount of pluc-Mash1 (100 ng) and pRL-CMV (0.2 ng) in the presence or absence of increasing amounts of expression plasmid for siRNA against human LMO3 (100 or 400 ng). Forty-eight hours after transfection, cells were lysed and their luciferase activities were measured. (TIF) [file pone.0019297.s001.tif]

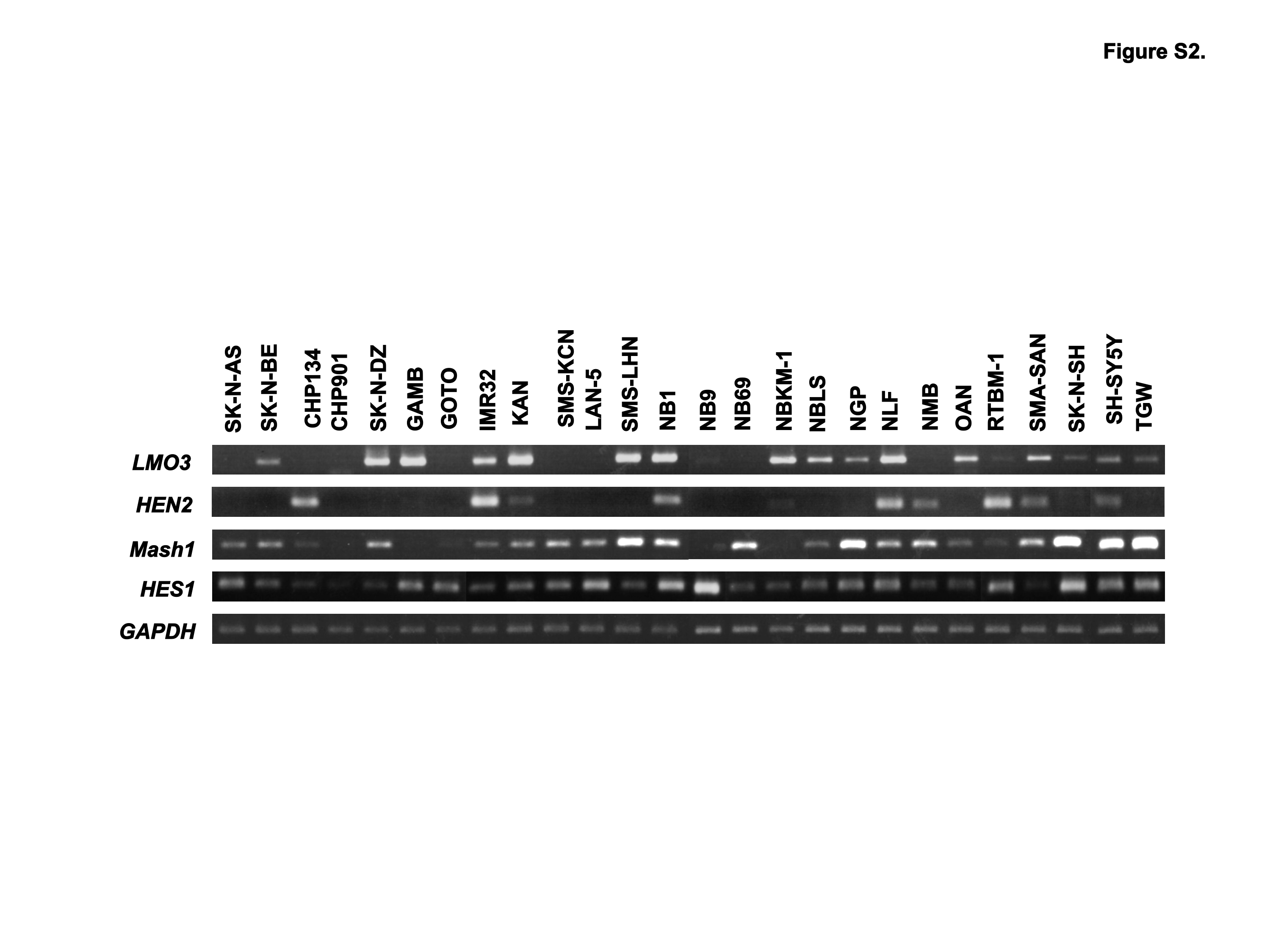

Supplement: Figure S2 — Expression of LMO3 , HEN2 , Mash1 or HES1 in neuroblastoma cell lines. Semiquantitative RT-PCR analysis for expression of LMO3, HEN3, Mash1 or HES1 in neuroblastoma cell lines is performed under linear amplification conditions. Expression of GAPDH is shown as a control. (TIF) [file pone.0019297.s002.tif]

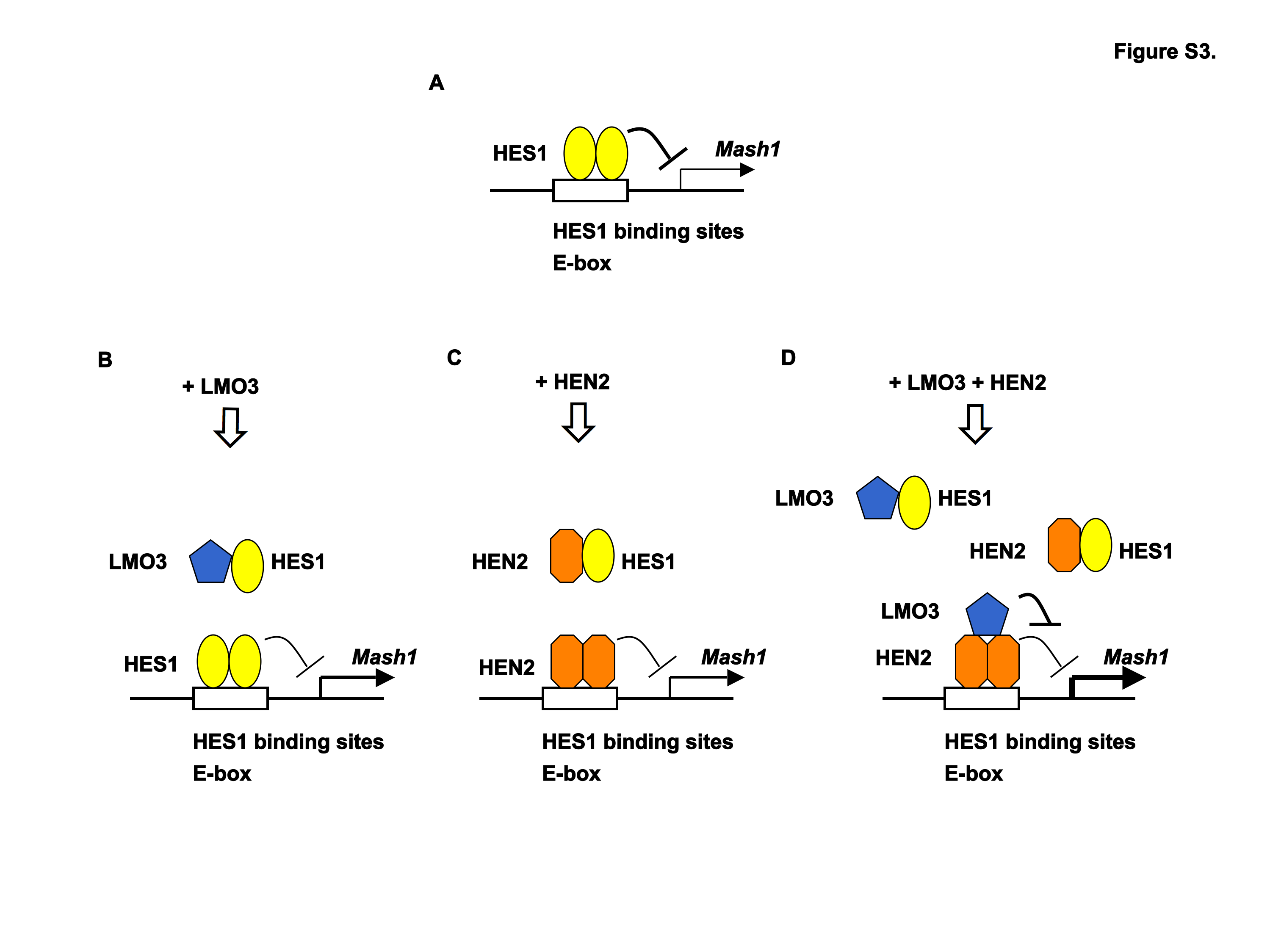

Supplement: Figure S3 — Model for LMO3 and HEN2 cooperation in transcriptional regulation of Mash1 in Neuroblastoma. (A) HES1 binds to HES1 binding sites and E- box on Mash1 promoter and represses Mash1 transcription. (B) LMO3 inhibits recruitment of HES1 onto HES1-binding sites and E-box on Mash1 promoter by forming complex with HES1, and thereby inducing the expression of Mash1. (C) HEN2 interferes with recruitment of HES1 onto HES1-binding sites and E-box on Mash1 promoter by forming complex with HES1 and competing with HES1 in binding to these sites. HEN2 also represses Mash1 transcription but the inhibitory effects are weaker than that of HES1, and so up-regulating transcription of Mash1. (D) LMO3 promotes recruitment of HEN2 onto HES1-binding sites and E-box on Mash1 promoter by forming complex with HEN2 but inhibits negative effects of HEN2 on Mash1 promoter. Furthermore, LMO3 inhibits recruitment of HES1 onto HES1-binding sites and E-box on Mash1 promoter, and so Mash1 may be more highly expressed. (TIF) [file pone.0019297.s003.tif]
