# Supplementary material for: Oncogenic LMO3 Collaborates with HEN2 to Enhance Neuroblastoma Cell Growth through Transactivation of Mash1
Source: PLoS One. 2011 May 5;6(5):e19297. doi: 10.1371/journal.pone.0019297 (PMC3088666; doi:10.1371/journal.pone.0019297)
Supplement: Table S1 — Correlation between expression of LMO3 or Mash1 and other prognostic factors (Student's t-test). (PDF) [file pone.0019297.s004.pdf]

Table S1. Correlation between expression of *LMO3* or *Mash1* and other prognostic factors (Student's t-test).

Factor	No.	*LMO3* expression		*Mash1* expression	
		Mean ± SEM	*p*-value	Mean ± SEM	*p*-value
Age					
<1 year	41	0.76 ± 0.16	0.036	5.16 ± 1.09	0.810
≥1 year	59	1.51 ± 0.31		4.75 ± 1.28	
Tumor stage					
1, 2, 4s	44	0.91 ± 0.23	0.174	2.32 ± 0.60	0.004
3, 4	56	1.43 ± 0.30		6.96 ± 1.44	
TrkA expression					
Low	45	1.93 ± 0.40	0.003	5.70 ± 1.71	0.398
High	53	0.60 ± 0.09		4.11 ± 0.76	
MYCN copy number					
Amplified	17	2.51 ± 0.69	0.040	10.47 ± 3.94	0.112
Single	83	0.93 ± 0.18		3.78 ± 0.64	
Tumor origin					
Adrenal	53	1.51 ± 0.33	0.083	5.96 ± 1.46	0.196
Others	47	0.84 ± 0.19		3.75 ± 0.86	
Shimada classification					
Favorable	61	0.92 ± 0.18	0.082	4.36 ± 0.85	0.450
Unfavorable	27	2.01 ± 0.58		6.40 ± 2.54	
