# Supplementary material for: Oncogenic LMO3 Collaborates with HEN2 to Enhance Neuroblastoma Cell Growth through Transactivation of Mash1
Source: PLoS One. 2011 May 5;6(5):e19297. doi: 10.1371/journal.pone.0019297 (PMC3088666; doi:10.1371/journal.pone.0019297)
Supplement: Table S2 — Univariate and multivariate analyses of Mash1 and LMO3 mRNA expression as well as other prognostic factors in primary neuroblastomas. (PDF) [file pone.0019297.s005.pdf]

Table S2. Univariate and multivariate analyses of *Mash1* and *LMO3* mRNA expression as well as other prognostic factors in primary neuroblastomas

Factor	n	*p*-value	H.R.	C.I.
Age (≥1 year vs. <1 year)	59 vs. 41	0.011	3.2	(1.3, 9.5)
Stage (1, 2, 4s vs. 3, 4)	44 vs. 56	<0.001	7.3	(2.5, 30.9)
MYCN (amplified vs. single)	17 vs. 83	<0.001	9.7	(4.3, 21.9)
Origin (non-adrenal vs. adrenal)	47 vs. 53	0.002	3.9	(1.6, 10.6)
Mash1 expression (high vs. low)	30 vs. 70	0.048	2.2	(1.0, 4.8)
LMO3 expression (high vs. low)	26 vs. 74	0.012	2.9	(1.3, 6.6)
Age (≥1 year vs. <1 year)	59 vs. 41	0.006	3.5	(1.4, 10.5)
Mash1 expression (high vs. low)	30 vs. 70	0.024	2.5	(1.1, 5.5)
Stage (1, 2, 4s vs. 3, 4)	44 vs. 56	<0.001	6.7	(2.2, 29.1)
Mash1 expression (high vs. low)	30 vs. 70	0.559	1.3	(0.6, 2.8)
MYCN (amplified vs. single)	17 vs. 83	<0.001	9.7	(4.2, 22.3)
Mash1 expression (high vs. low)	30 vs. 70	0.093	2.0	(0.9, 4.4)
Origin (non-adrenal vs. adrenal)	47 vs. 53	0.002	3.8	(1.6, 10.3)
Mash1 expression (high vs. low)	30 vs. 70	0.064	2.1	(1.0, 4.6)
Age (≥1 year vs. <1 year)	59 vs. 41	0.014	3.0	(1.2, 9.1)
LMO3 expression (high vs. low)	26 vs. 74	0.015	2.9	(1.2, 6.5)
Stage (1, 2, 4s vs. 3, 4)	44 vs. 56	<0.001	7.0	(2.4, 29.7)
LMO3 expression (high vs. low)	26 vs. 74	0.021	2.7	(1.2, 6.3)
MYCN (amplified vs. single)	17 vs. 83	<0.001	8.4	(3.6, 19.5)
LMO3 expression (high vs. low)	26 vs. 74	0.213	1.7	(0.7, 4.1)
Origin (non-adrenal vs. adrenal)	47 vs. 53	0.003	3.6	(1.5, 9.9)
LMO3 expression (high vs. low)	26 vs. 74	0.021	2.6	(1.2, 5.8)
Mash1 expression (high vs. low)	30 vs. 70	0.022	2.6	(1.1, 5.7)
LMO3 expression (high vs. low)	26 vs. 74	0.006	3.4	(1.4, 7.8)

Footnotes; n: number of samples, H.R.: hazard ratio, C.I.: confidence interval.
